# Multi-tissue metabolomics analysis reveals susceptible factors for chemotherapy-induced hepatotoxicity in colorectal cancer patients

**DOI:** 10.3389/fphar.2025.1517446

**Published:** 2025-04-04

**Authors:** Huilin Xu, Mingming Li, Houshan Yao, Guoliang Chen, Jiani Chen, Xinyun Hou, Hong Yang, Chenghang Yu, Zeshuai Lin, Jiawei Zhu, Rong Wang, Shi Qiu, Xuan Liu, Zhipeng Wang, Xia Tao, Lei Liu

**Affiliations:** ^1^ Institutes of Biomedical Sciences and Intelligent Medicine Institute, Fudan University, Shanghai, China; ^2^ Department of Pharmacy, Second Affiliated Hospital of Naval Medical University, Shanghai, China; ^3^ Department of General Surgery, Second Affiliated Hospital of Naval Medical University, Shanghai, China; ^4^ National Institute of Parasitic Diseases, Chinese Center for Disease Control and Prevention (Chinese Center for Tropical Diseases Research), Key Laboratory of Parasite and Vector Biology, National Health Commission of the People’s Republic of China; WHO Collaborating Center for Tropical Diseases, Shanghai, China; ^5^ Traditional Chinese Medicine Resource and Technology Center, Shanghai University of Traditional Chinese Medicine, Shanghai, China; ^6^ Department of Traditional Chinese Medicine, Changzheng Hospital, Naval Medical University, Shanghai, China

**Keywords:** chemotherapy-induced hepatotoxicity, multi-omics analysis, capeox, endogenous susceptible biomarkers, metabolomics

## Abstract

**Amis:**

Chemotherapy-induced hepatotoxicity (CIH) is a significant concern in colorectal cancer (CRC) patients treated with the CAPEOX (capecitabine and oxaliplatin) regimen. Identifying predictive factors for CIH is crucial for clinical management.

**Patients and Methods:**

This study analyzed colorectal tissue (CRT), plasma, and urine samples from CRC patients. Differentially expressed metabolites (DEMs) across these tissues were integrated for multi-omics analysis, and predictive models for CIH susceptibility were developed. An independent set of 75 plasma samples was used for validation.

**Results:**

A total of 492 differentially expressed compounds were identified in samples from 63 CRC patients, including 105, 149, and 238 DEMs in CRT, plasma, and urine, respectively. Lipids and lipid-like molecules were predominant in all samples. Among these, urine samples exhibited the highest variability and provided the strongest predictive power for CIH susceptibility. Principal component analysis (PCA) effectively differentiated normal patients from those with CIH. The study revealed steatosis as the primary pathological feature of CIH, with disrupted lipid metabolism emerging as a key characteristic. Predictive models constructed from multi-tissue metabolites profile exhibited high accuracy, with the plasma model achieving an AUC of 0.933 in external validation set. Our study underscores the importance of individual metabolic variations in CIH susceptibility, reflecting the complex interplay of genetic, environmental, and lifestyle factors.

**Conclusion:**

This study emphasizes the critical role of alterations in lipid, polyamine, and purine metabolism, as well as impaired tissue repair mechanisms, were identified as key endogenous factors underlying CIH susceptibility. The developed predictive models demonstrate potential for clinical application in assessing CIH risk in CRC patients undergoing CAPEOX chemotherapy.

## 1 Introduction

CAPEOX (capecitabine + oxaliplatin) is the most recommended and commonly used chemotherapy regimen for various solid tumors, including colorectal cancer (CRC) (Bray et al., 2018). However, due to its cytotoxicity, CRC patients experience a variety of adverse effects after chemotherapy, including hepatotoxicity, with a prevalence of 30%–47% (McWhirter et al., 2013; Li et al., 2021a). The causes of chemotherapy-induced hepatotoxicity (CIH) vary among liver failure, cholestasis, steatosis, ductal damage fibrosis, osteoporotic hepatitis, and venous obstruction (McDonald and Tirumali, 1984; Gangi and Lu, 2020a). Among these, steatosis—a condition marked by lipid accumulation within hepatocytes—is recognized as an early stage of nonalcoholic fatty liver disease (NAFLD). The mechanisms underlying lipid accumulation include excessive free fatty acid (FFA) import, reduced hepatic export, and impaired FFA oxidation. If unresolved, steatosis can progress to steatohepatitis, characterized by liver cell injury and inflammation, which may ultimately lead to fibrosis. Another distinct CIH manifestation is sinusoidal obstruction syndrome (SOS), caused by damage to endothelial cells lining liver sinusoids. Unlike steatosis, SOS primarily affects vascular integrity without directly impairing hepatocyte function.

Effective prediction and prevention of CIH require reliable biomarkers and a deeper understanding of the underlying molecular mechanisms. Significant research has identified potential metabolite biomarkers for CIH, offering promising candidates for further investigation. Notably, elevated levels of sphingosine-1-phosphate (S1P) and lysophosphatidylcholine (LysoPC) have been observed in patients undergoing cisplatin chemotherapy and are correlated with liver injury (Chen J. et al., 2018; Ji et al., 2020). Similarly, increased concentrations of several bile acids, including taurocholic acid and glycochenodeoxycholic acid, have been associated with CIH in patients receiving irinotecan (Aubrecht et al., 2013; Yang et al., 2014). These findings underscore the potential of metabolite biomarkers to enhance patient safety by enabling early detection of CIH and optimizing CRC treatment strategies.

Current research on chemotherapy-induced hepatotoxicity (CIH) faces two primary limitations. First, most studies investigating biomarkers of chemotherapy-related adverse events (CRAE) focus on drug metabolism alone, neglecting the impact of inter-individual differences, which are known to be crucial determinants of the occurrence and severity of chemotherapy-related side effects, including CIH (Gelibter et al., 2019). In contrast, metabolomics provides a holistic diagnostic approach by analyzing low molecular weight organic compounds in various biological fluids and tissues, such as blood, urine, saliva, and tissue samples (Bujak et al., 2015). Unlike the genome, transcriptome, and proteome, the metabolome reflects the end product of metabolism, offering a more direct link to the phenotype (Weiss and Kim, 2011; Everett et al., 2019; Rinschen et al., 2019). Second, many studies rely on a single type of tissue sample for biomarker screening, limiting the ability to assess the relative contributions of different tissue types. This narrow focus often neglects the potential influence or correlation of CIH-related features across tissues, thereby restricting a comprehensive understanding of the molecular mechanisms underlying CIH. Addressing these limitations through a multi-tissue metabolomics approach can enhance the ability to identify reliable predictive markers and provide deeper insights into the complex molecular pathways driving CIH.

Multiple omics integrative analysis methods, such as NNF (Zhang et al., 2011; Zhang et al., 2012) and iCluster (Shen et al., 2009; Shen et al., 2012) have been developed to analyze complex biological datasets. However, these methods often exhibit limited tolerance for missing data, which can compromise their effectiveness. The multi-omics factor analysis (MOFA) algorithm can handle missing values and detect sample abnormalities (Argelaguet et al., 2018; Argelaguet et al., 2020). MOFA was demonstrated to have a better classification ability than SNF and iClusterBayes (Zheng et al., 2023).

In this study, we utilized MOFA to integrate metabolomic data derived from colorectal tissue (CRT), plasma, and urine samples. This comprehensive multi-omics approach aimed to elucidate the molecular mechanisms underlying CIH susceptibility and develop predictive models to identify potential biomarkers for assessing CIH susceptibility.

## 2 Materials and methods

### 2.1 Patient selection and sample collection

All patients were enrolled from a clinical trial conducted at Shanghai Changzheng Hospital from June 2018 to December 2021 (registration number: NCT03030508). The inclusion criteria for CRC patients were (1) age over 18 years, (2) biopsy diagnosis of CRC, (3) first-time CRC patients, and (4) no preoperative treatment with antineoplastic drugs. Written informed consent was obtained from all patients before sample collection. The Ethics Committee of Shanghai Changzheng Hospital approved the study. Clinical information, including age (in years), gender (female or male), and body mass index (BMI), were also collected.

All patients received oxaliplatin (0.16–2 g/d) intravenously on day one and oral capecitabine (1.5 g/d) during the first 2 weeks. All patients have completed at least four chemotherapy cycles. Patients were followed up with relevant clinical data during chemotherapy and then assessed for the occurrence and severity of CIH according to CTCAE5.0 criteria. Hepatotoxicity was clinically determined by alanine aminotransferase (ALT) or aspartate aminotransferase (AST) over five times ULN (Upper Limit of Normal) during the chemotherapy after the surgery (Meunier and Larrey, 2019). Elevations in AST and ALT are well-established indicators of hepatocellular injury. For instance, acetaminophen-induced hepatotoxicity in animal models has been shown to cause marked increases in ALT and AST levels within 24 h (McGill and Jaeschke, 2019). Patients were categorized into a normal group and a CIH group based on CIH occurrence.

Urine samples from enrolled CRC patients were collected in lyophilized tubes one to 2 days before surgery; 5 mL of blood samples from enrolled patients were collected in EDTA anticoagulation tubes, centrifuged (2000 g, 15 min, 4°C), and then blood was collected and divided into lyophilized tubes (400 μL/freezing tube); CRT was collected during the surgery (more than 10 cm from the tumor tissue). All samples were stored in a −80°C refrigerator within 2 h after acquisition. For detailed sample processing information, please refer to our previously published works (Li et al., 2021b; Yao et al., 2022a).

### 2.2 Metabolomics analysis and data preprocessing

Untargeted metabolomics was employed to screen 63 patients, aiming to identify features related to CIH and construct a predictive. Subsequently, pseudo-targeted metabolomics was applied to an additional 75 patients for model validation.

Mass spectrometry (MS) data were analyzed using the ProFinder program (version b8.0, Agilent Technologies). After fusion alignment, spectral features were recursively extracted based on retention time (RT), mass-to-charge ratio (m/z), and spectral area. Spectral features generated by internal standards, noise, and column bleeding are removed from the data set. Features resulting from internal standards, noise, and column bleeding were excluded. The processed results were manually validated and transferred to the Mass Profiler Professional program for further analysis (Agilent Technologies). The 80% rule was applied to reduce data dimensionality and avoid missing values (Bijlsma et al., 2006). Spectral features were normalized to the sum of the intensities for each sample. Metabolite detection utilized both positive and negative ionization modes, with peak area normalization applied to eliminate duplicates across modes. Detailed methods for compound identification are available in our previous studies (Li et al., 2021b; Yao et al., 2022a).

### 2.3 Statistical and multi-omics analysis

*After acquiring MS data, log2 transformation and normalization were applied. Differential expression analysis was then conducted using the R package limma* (Ritchie et al., 2015), to compare the CIH and normal groups, adjusting for clinical covariates. Metabolites identified as differentially expressed (DEMs) with a significance threshold of p < 0.05 were selected as inputs for multi-omics analysis. This pre-selection step aimed to reduce noise and identify metabolites that differentiate CIH-susceptible patients from those in the normal group. PCA was performed on DEMs to visualize the difference between the CIH and normal groups with R package “FactoMineR” and “factoextra”.

For integrative analysis, the MOFA algorithm was utilized for integrative analysis through R package MOFA2 (Argelaguet et al., 2018). Key parameters included maxiter set to 5,000, convergence_mode set to “slow,” and other settings left at default values. From a biological standpoint, the LF1 weights reflect how strongly each metabolite contributes to this latent factor, thereby highlighting key metabolic disturbances relevant to CIH. This integrative approach not only consolidates signals across multiple biofluids and tissues but also help underscores the complex interplay of pathways driving hepatocellular injury.

### 2.4 Pathway enrichment analysis

The DEMs were subjected to pathway enrichment analysis through the Reactome website (Gillespie et al., 2022) with HMDB ID. The Reactome classifications were utilized for pathway classification. Pathways belonging to the disease category were excluded in the subsequent analysis. Pathways with *p* < 0.05 and enriched metabolites number ≥3 were considered for further research. The MOFA weight and log2FC value were utilized to determine the pathway weight and direction as described in our previous studies (Li et al., 2021b; Yao et al., 2022a).

### 2.5 Correlation analysis

Pearson correlation analysis was conducted on DEMs that met the following criteria: (1) a higher absolute MOFA weight and (2) inclusion in the top three most significant pathways of plasma, urine, and CRT. Compounds with an absolute correlation coefficient r2 ≥ 0.4 and the highest number of connections to other DEMs were identified as candidate biomarkers. The correlation networks were visualized with Gephi (version v0.9.2).

### 2.6 Predictive model construction

To further filter candidate biomarkers, we employed the random forest method using the R package “randomForest,” with the process repeated 1,000 times for enhanced robustness. Features that appeared at least 500 times in the overall ranking were selected as final biomarkers. The dataset was randomly split into training and test sets at a ratio of 7:3, and a multivariate logistic regression model was constructed final biomarkers. The predictive performance of the model was evaluated by plotting the ROC curve using the R package plotROC.

### 2.7 Mendelian Randomization

To overcome sample size limitations and strengthen causal inference between metabolic alterations and CIH, we applied a two-sample Mendelian Randomization (MR) analysis. We selected compounds with corresponding GWAS IDs from the IEU Open GWAS project to explore potential causal relationships with AST and ALT levels. The SNP–metabolite associations (exposure) were obtained from metabolomics GWAS data, while the SNP–CIH associations (outcome) were derived from our clinical dataset. Our primary analysis employed the inverse variance weighted (IVW) random-effects method, which estimates the causal effect by meta-analyzing the Wald ratios (i.e., the SNP’s effect on CIH divided by its effect on the metabolite). To address potential violations of the IV assumptions, we further performed MR-Egger regression—which allows for horizontal pleiotropy—and the weighted median method, robust even when up to 50% of the IVs are invalid. Heterogeneity was evaluated using Cochran’s Q test and leave-one-out analysis was conducted to ensure that no single SNP disproportionately influenced the overall estimate.

## 3 Results

### 3.1 Patient characteristics

**FIGURE 1:**
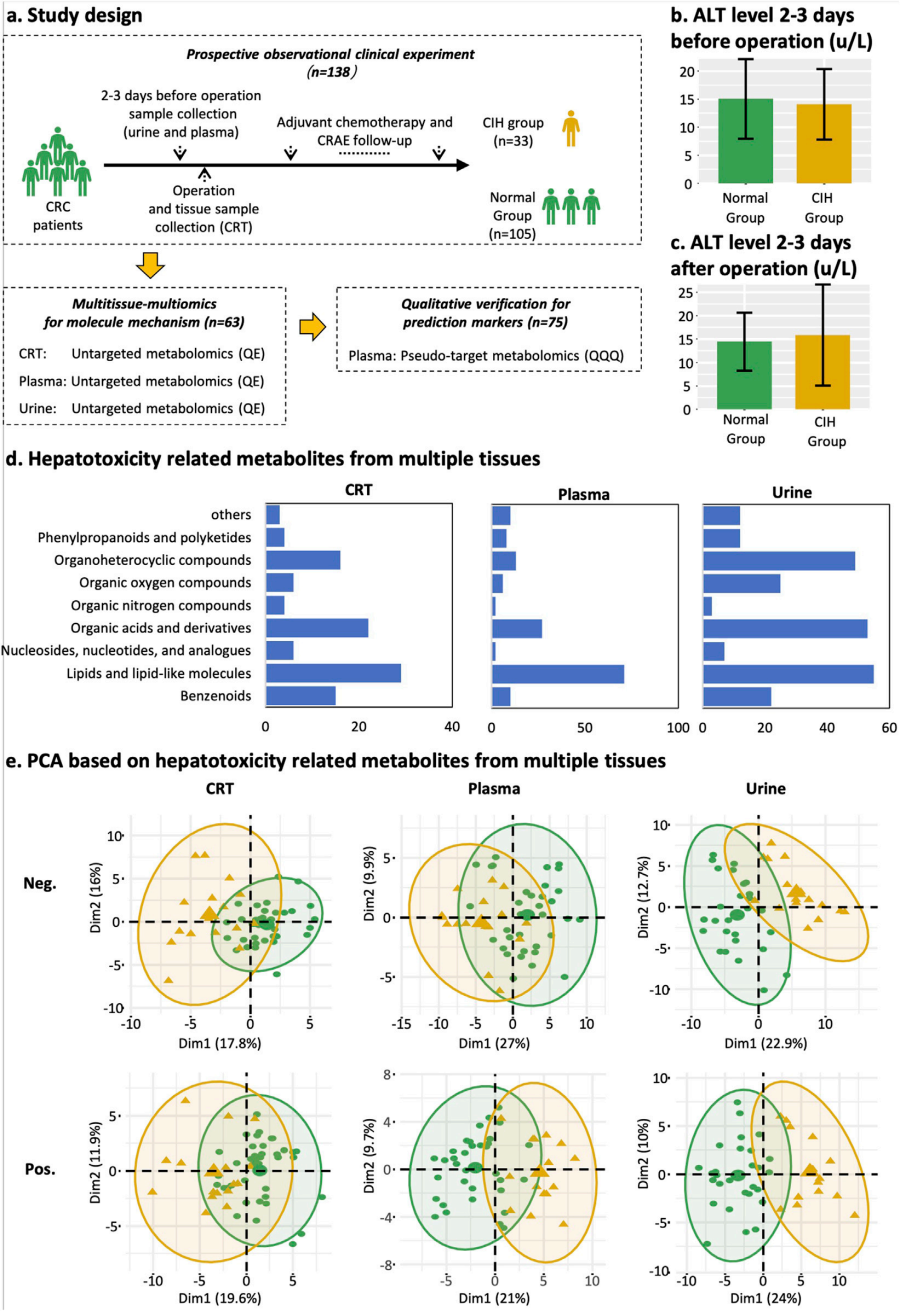
Study design, classification of differentially expressed metabolites, and PCA plot. **(a)** Study design and sample collection time point of each dataset. Yellow represents the CIH group, and green represents normal groups. **(b)** Mean ALT level of normal group and CIH group 2–3 days before the operation. **(c)** Mean ALT level of normal group and CIH group 2–3 days after the operation. **(d)** Classification and count of CIH-related compounds in CRT, plasma, and urine samples from left to right. The X-axis represents the counts of CIH-associated compounds. The Y-axis represents HMDB classifications. **(e)** PCA score plot of CRT, plasma, and urine metabolic profiling. Yellow means the CIH group. Green indicates the normal group. Abbreviations: Pos, positive ion modes; Neg, negative ion modes.

**FIGURE 2:**
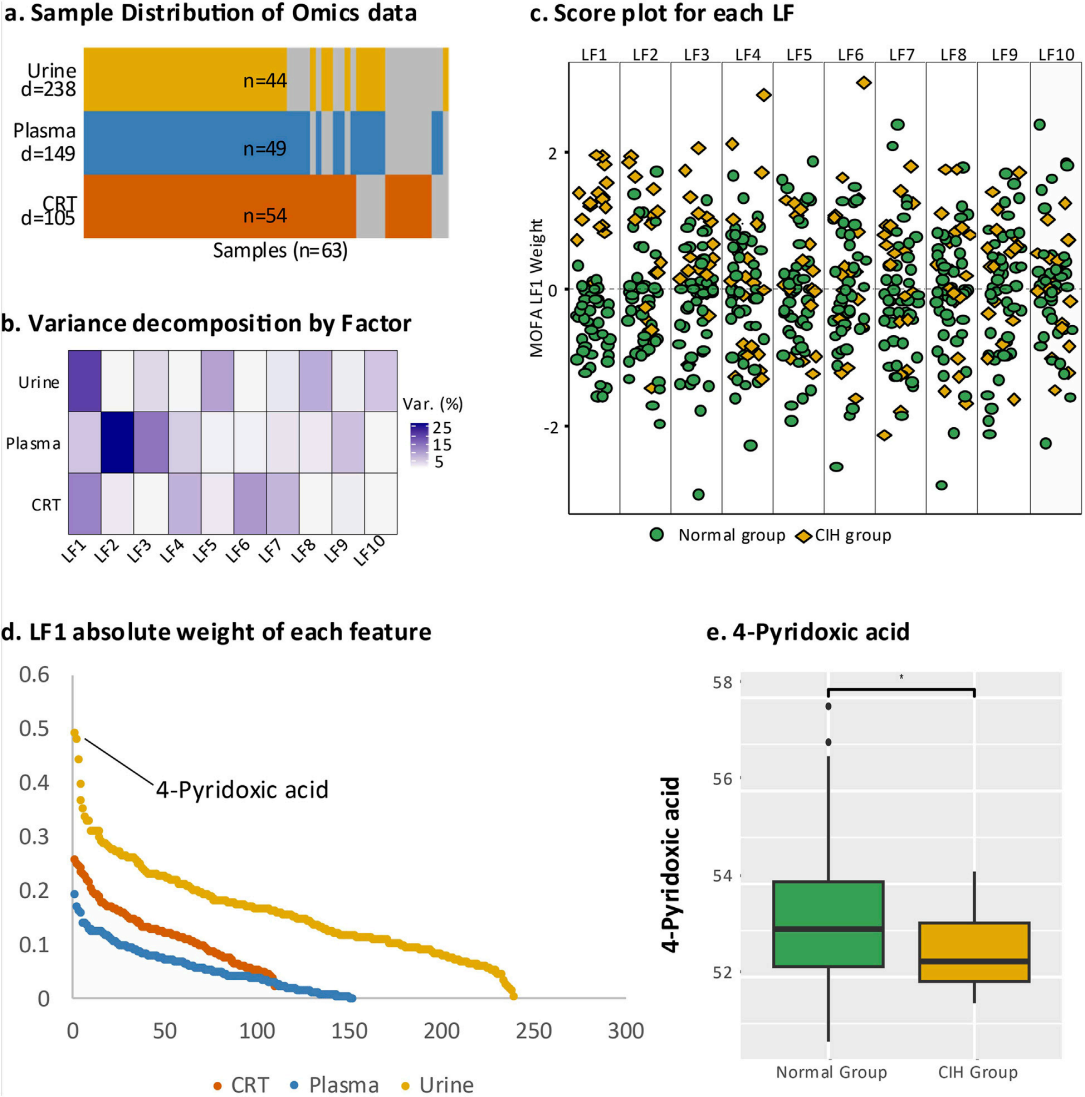
Multi-omics analysis of endogenous DEMs. **(a)** Overall representation of the three sample types, where “N” at the bottom indicates the total number of samples and “D” represents the number of DEMs identified in urine, plasma, and CRT samples. Yellow denotes urine samples, blue indicates plasma samples, and orange represents CRT samples; grey areas indicate missing data. **(b)** The contribution of latent factors in explaining the variation across urine, plasma, and CRT samples. **(c)** Beeswarm plot illustrating the discriminative ability of each latent factor, with green representing the normal group and yellow representing the CIH group. **(d)** Scatter plot displaying the top-ranking absolute values of LF1 weights. **(e)** Boxplot comparing 4-pyridoxic acid levels between the normal and CIH groups, with statistical significance annotated (e.g., asterisks) to indicate the differences between groups.

63 patients were enrolled in this study, with 19 classified into the CIH group and 44 into the normal group (Figure 1A). Among these, CRT samples were obtained from 54 patients, plasma samples from 49 patients, and urine samples from 44 patients (Figure 2A). The normal patient group consisted of 44 individuals, with an equal distribution of 22 patients aged over 60 years and 22 patients aged under 60 years. While the CIH group has 11 patients under the age of 60 and 8 patients were over 60. The mean age in the normal group was 56.5, while the median age was 56. The mean age for CIH group patient was 59.1, while the median age was 59.6 (Table 1). The results suggest that a higher BMI (>24) was potentially related to CIH, as more patients in the CIH group had a BMI ≥24 (15.8% vs. 14.3%) compared to what was in the normal group (22.2% vs. 47.6%). Conversely, no clear association was observed between CIH and age or gender, as the proportions of patients aged over 60 or male/female were similar between the two groups (Table 1).

**TABLE 1:** Patient characteristics.

Clinical features	Classification	Normal group	CIH group
Age	<60	22 (51,50.09)	11 (50.49.91)
	≥60	22 (67,68.13)	8 (66,65.5)
Gender	Male	30 (47.6%)	12 (19.0%)
	Female	14 (22.2%)	7 (11.1%)
BMI	<24	30 (47.6%)	9 (14.3%)
	≥24	14 (22.2%)	10 (15.8%)

Notes:

For Age, the numbers in parentheses represent the median and mean.

%means the proportion of cell count in the whole group.

Before and after the operation, the alanine aminotransferase (ALT) levels in patients from both the normal group and the CIH group remained within the normal clinical range of 5–40 U/L. Specifically, the ALT value was under 20 U/L in the normal group (Figure 1B) and 25 U/L in the CIH group (Figure 1C). This indicates that patients did not exhibit hepatotoxicity prior to chemotherapy, ensuring that any hepatotoxic effects observed during the study can be attributed specifically to the chemotherapy regimen rather than pre-existing liver dysfunction.

Additionally, plasma samples from another 75 patients were collected for model validation. This decision was based on the ease of plasma collection and its significant clinical utility (Figure 1A).

### 3.2 Categories of differentially expressed endogenous metabolites

A total of 5,028 compounds were subjected to differential analysis after compound identification and preprocessing. This process led to the identification of 105, 149, and 238 DEMs in CRT, plasma, and urine samples, respectively (Figure 2A). PCA plots revealed that these DEMs, identified in both positive and negative ionization modes, effectively distinguished the normal group from the CIH group (Figure 1E). Taxonomic classification of the DEMs using the Human Metabolome Database (HMDB) revealed nine categories with lipids and lipid-like molecules emerging as the most abundant and significantly altered class across CRT, plasma, and urine samples (Figure 1D).

### 3.3 Identification of top-ranking endogenous DEMs

The integrative analysis of differentially expressed metabolites (DEMs) from CRT, plasma, and urine using multi-omics factor analysis (MOFA) identified ten latent factors (LFs) that collectively explained the variation in our dataset. LF1 demonstrated the highest explanatory power for urine (21%) and CRT (16%) samples, while LF2 contributed the most to the variation in plasma (36%) (Figure 2B). Notably, LF1 showed the strongest discriminatory ability between the normal and CIH groups (Figure 2C), underscoring its clinical relevance.

We subsequently leveraged the weight values derived from LF1 for downstream analysis to pinpoint the most critical metabolic perturbations associated with CIH. Among the endogenous DEMs, 4-pyridoxic acid in urine exhibited the highest absolute weight value (0.439, *p* < 0.000016) and was significantly downregulated in the CIH group (Log2FC = −0.779, *p* < 0.046) (Figures 2D,E). In plasma, 2-Phenylbutyric acid had a higher absolute weight value (0.169, *p* < 0.016), while in CRT, 3-Hydroxybutyrate showed a higher absolute weight value (0.201, *p* < 0.002).

### 3.4 Integrative pathway enrichment analysis

**FIGURE 3:**
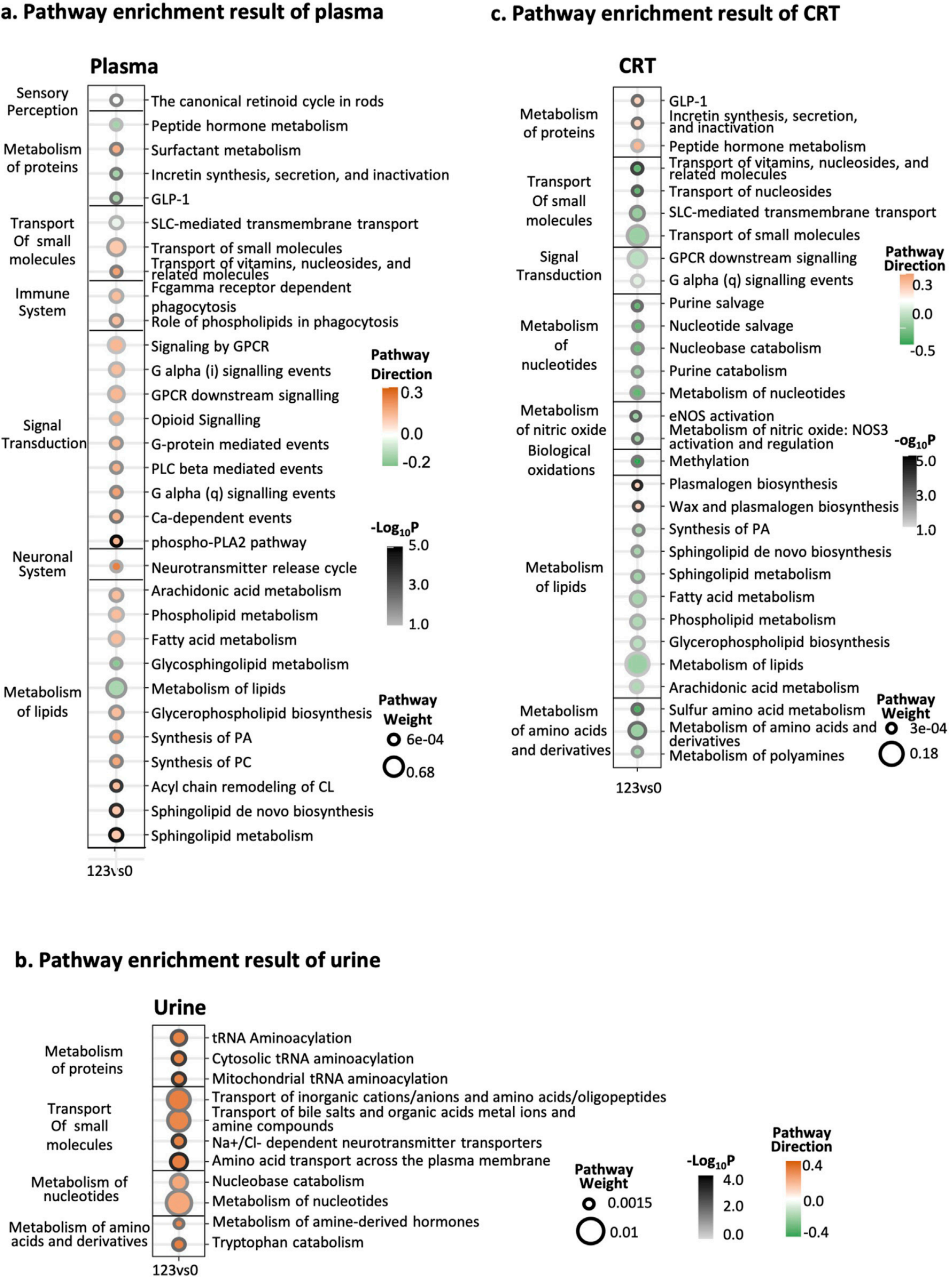
Integrative pathway enrichment results. **(a)** Pathway enrichment results of plasma samples. **(b)** Pathway enrichment results of urine samples. **(c)** Pathway enrichment results of CRT samples. The Y-axis on the left side of the graph is the pathway’s classification, and the pathway’s name is on the right side. In the X-axis, 123 represents the CIH group, while 0 represents the normal group. The circle size means the weight of the pathway. The circle color represents the direction of the pathway. The darkness of the circle border represents the significance of the pathway.

Building on these LF1-driven insights, we re-evaluated pathway importance and direction using both MOFA weights and log2FC values (Figure 3). In plasma, most pathways were upregulated, with lipid metabolism and signal transduction carrying the highest weights; the top three significant pathways were “phospho-PLA2 pathway,” “sphingolipid metabolism,” and “Sphingolipid *de novo* biosynthesis” (Figure 3A). In urine, all pathways were upregulated, notably “amino acid transport across the plasma membrane,” “mitochondrial aminoacylation,” and “sodium/chloride-dependent neurotransmitter transporters” (Figure 3B). Meanwhile, CRT samples showed a predominantly downregulated trend, except for pathways linked to protein and lipid biosynthesis, including “plasmalogen biosynthesis,” “wax and plasmalogen biosynthesis,” and “transport of vitamins, nucleosides, and related molecules” (Figure 3C). The detailed pathway enrichment results was in Supplementary Table 2.

### 3.5 Network visualization of critical DEMs

The correlation analysis revealed that lipids such as phosphatidylethanolamine (PE), phosphatidylcholine (PC), sphingomyelin (SM), sphingosine-1-phosphate (S1P), and lysophosphatidic acid (LPA) exhibited the largest node size, indicating their high correlation with other metabolites in the network, particularly in plasma samples.

**FIGURE 4:**
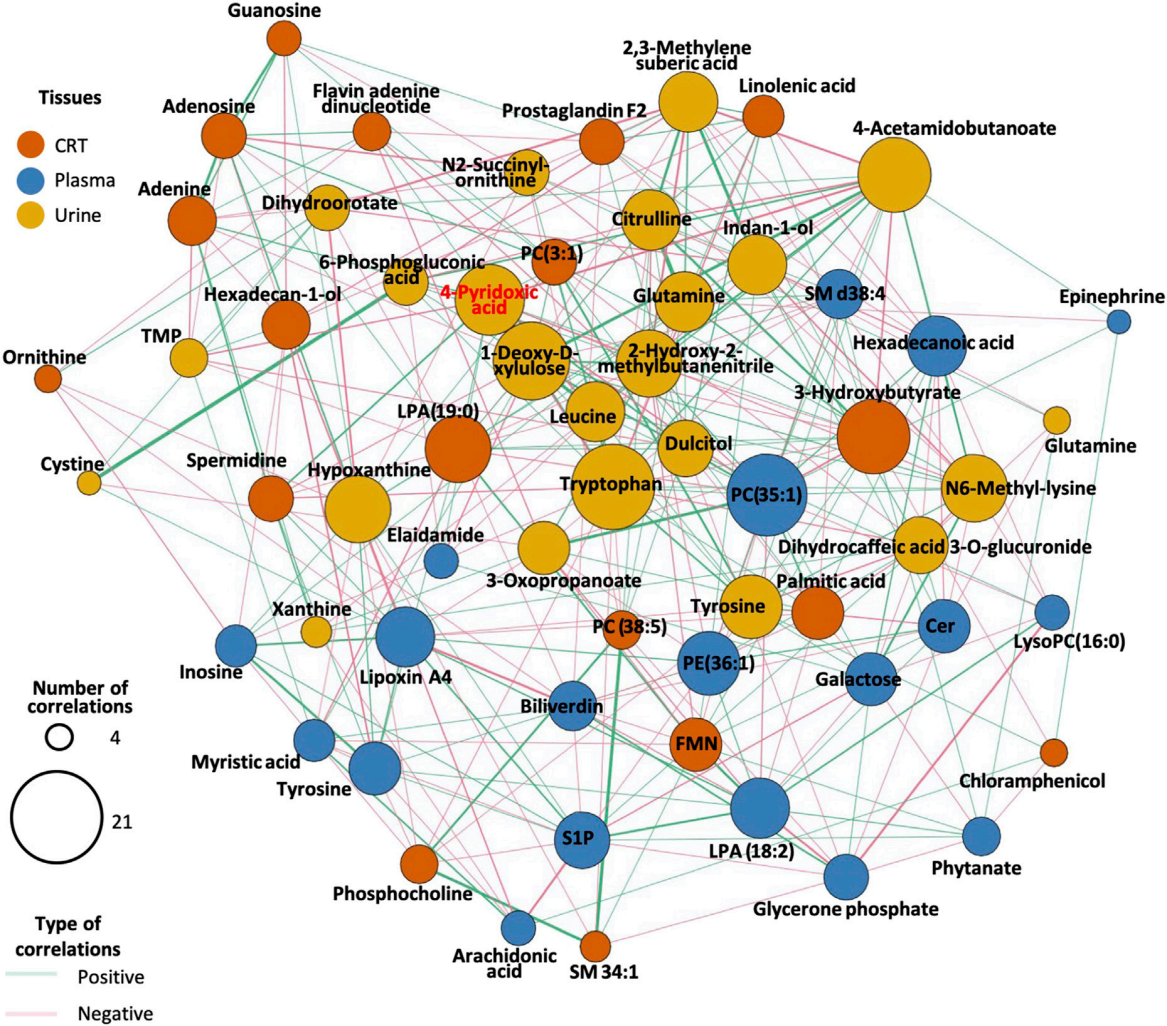
Correlation network diagram for endogenous DEMs. The size of the circles in the graph represents the number of DEMs associated with the compound. The line’s color between the circles indicates whether the two compounds are positively or negatively correlated. The color of the circles in the diagram represents the different classifications of the compounds.

In urine samples, metabolites such as 4-pyridoxic acid, tryptophan, leucine, 1-deoxy-D-xylulose, hypoxanthine, and 4-acetamidobutanoate exhibited larger node sizes compared to other differentially expressed metabolites (DEMs), suggesting their central role in the metabolic network (Figure 4). The metabolites have the most related DEMs were selected as candidate biomarkers for predictive models. Full candidate biomarkers identified was shown in Table 2 and there corresponding enriched pathways was shown in Supplementary Table S2.

**TABLE 2:** Candidate biomarkers.

Name	HMDB	KEGG	Log2FC	Absolute weight	P	Tissue
3-Hydroxybutyrate	HMDB0000011	C01089	−0.3518	0.2015	0.0016	CRT
Flavin adenine dinucleotide	HMDB0001520	C00061	−0.4838	0.1242	0.0024	CRT
Hexadecan-1-ol	HMDB0003424	C00823	0.2969	0.1441	0.0084	CRT
Adenine	HMDB0000034	C00147	−0.3867	0.1872	0.0031	CRT
Spermidine	HMDB0001257	C00315	−0.2469	0.1144	0.0251	CRT
Adenosine	HMDB0000050	C00212	−0.5229	0.1661	0.0007	CRT
Prostaglandin F2	HMDB0001139	C00639	−0.3912	0.1459	0.0184	CRT
PE (36:1)	HMDB0008992	C00350	−0.2684	0.0394	0.0052	plasma
Lipoxin A4	HMDB0004385	C06314	−0.1637	0.0064	0.0326	plasma
Palmitic acid	HMDB0000220	C00249	0.2359	0.1234	0.0004	plasma
S1P	HMDB0000277	C06124	−0.2274	0.0164	0.0273	plasma
Tyrosine	HMDB0000158	C00082	−0.1974	0.1763	0.0014	plasma
Cer	HMDB0004949	C00195	−0.2632	0.0257	0.0014	plasma
Galactose	HMDB0000143	C00984	0.1970	0.0429	0.0047	plasma
Tryptophan	HMDB0000929	C00078	0.2960	0.1398	0.0001	urine
1-Deoxy-D-xylulose	HMDB0001292	C06257	0.5156	0.3271	0.0001	urine
4-Acetamidobutanoate	HMDB0003681	C02946	0.4300	0.3087	0.0011	urine
4-Pyridoxic acid	HMDB0000017	C00847	−0.7794	0.4394	0.0000	urine
Hypoxanthine	HMDB0000157	C00262	−0.2985	0.0869	0.0034	urine
2-Hydroxy-2-methylbutanenitrile	HMDB0060309	C18796	0.4746	0.2832	0.0002	urine
N6-Methyl-lysine	HMDB0002038	C02728	0.8441	0.3952	0.0000	urine
Tyrosine	HMDB0000158	C06420	0.3572	0.1763	0.0014	urine
Indan-1-ol	HMDB0059601	NA	−0.6149	0.3079	0.0000	urine
Leucine	HMDB0000687	C00123	0.3134	0.1991	0.0036	urine
Glutamine	HMDB0003423	C00819	−0.5436	0.3084	0.0015	urine
2,3-Methylene suberic acid	HMDB0059779	NA	−0.6099	0.3660	0.0000	urine
Citrulline	HMDB0000904	C00327	−0.4283	0.3060	0.0079	urine
Dihydrocaffeic acid 3-O-glucuronide	HMDB0041720	NA	−0.8092	0.2712	0.0000	urine
Galactitol	HMDB0000107	C01697	−0.9515	0.3092	0.0002	urine
3-Oxopropanoate	HMDB0011111	C00222	0.2137	0.0898	0.0096	urine

Notes:

“Name” column represents the metabolites name.

“HMDB” column represents the HMDB, database ID, of the metabolite.

“KEGG” column represents the KEGG, database ID, of the metabolite.

“logFC” column represents the log2FC, value from differentially analysis using limma package. It was calculated between patient vs. control, which means log2(Patient/Control).

“Absolute weight” column represents the absolute value of MOFA, weight.

“P” column represents the adjusted p-value of the metabolites from differentially analysis using limma package.

“Tissue” column represents tissue source of the metabolite.

### 3.6 Performance of predictive models

**FIGURE 5:**
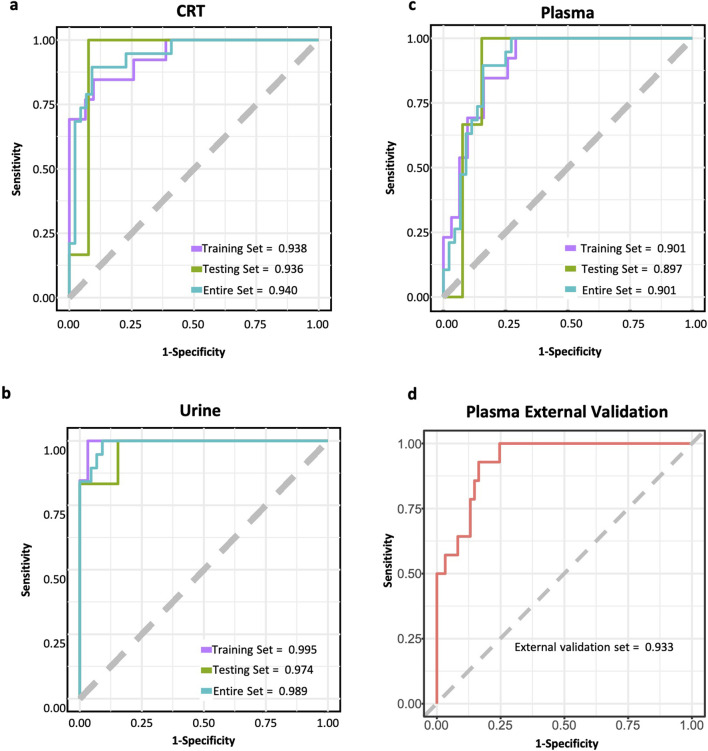
ROC curves of CIH predictive models. **(a)** ROC curve of CRT sample predictive model. **(b)** ROC curve of urine sample predictive model. **(c)** ROC curve of plasma sample predictive model. **(d)** ROC curve of the external test set for plasma sample predictive model. The purple line represents the ROC curve for the training set. The green line represents the ROC curve for the test set. The blue line represents the ROC curve for the whole dataset. The red line represents the ROC curve for the external test set.

In CRT samples, six metabolites—3-Hydroxybutyrate (BHB), Flavin adenine dinucleotide (FMN), adenosine, adenine, hexadecan-1-ol, and spermidine—were selected for predictive model construction based on their high frequency in the random forest models (Table 3). The predictive model achieved an area under the curve (AUC) of 0.938 for the training set and 0.936 for the test set, with an overall predictive accuracy of 0.940 across the entire dataset (Figure 5A).

For urine samples, the selected metabolites included indan-1-ol, 4-pyridoxic acid, N6-Methyl-lysine, dihydrocaffeic acid 3-O-glucuronide, and 1-deoxy-D-xylulose (Table 3). The AUC of training and test sets were 0.995 and 0.974, respectively. The predictive power of the whole dataset was 0.989 (Figure 5B).

**TABLE 3:** Information on biomarkers in CRT, plasma, and urine samples.

Name	Freq	P	OR_95CI	Tissue
3-Hydroxybutyrate	939	0.014	0.083 (0.009–0.506)	CRT
Flavin adenine dinucleotide	899	0.027	0.187 (0.035–0.73)	CRT
Adenosine	862	0.012	0.095 (0.012–0.486)	CRT
Adenine	845	0.036	0.133 (0.016–0.768)	CRT
Hexadecan-1-ol	650	0.013	14.396 (2.098–147.812)	CRT
Spermidine	581	0.084	0.149 (0.015–1.188)	CRT
Indan-1-ol	1,000	0.003	0.0001 (0–0.001)	urine
4-pyridoxic acid	823	0.001	0.004 (0–0.055)	urine
N6-Methyl-lysine	676	0.0001	302.89 (20.80–14427.55)	urine
Dihydrocaffeic acid 3-O-glucuronide	514	0.006	0.012 (0–0.139)	urine
1-Deoxy-D-xylulose	513	0.001	212.78 (12.41–8282.04)	urine
Palmitic acid	1,000	0.003	5,514.87 (41.10–5430520.72)	plasma
PE (36:1)	997	0.038	0.052 (0.002–0.659)	plasma
Cer	891	0.041	0.048 (0.002–0.767)	plasma
S1P	787	0.028	0.073 (0.005–0.64)	plasma
Galactose	709	0.011	180.817 (4.701–16053.254)	plasma

Notes:

“Name” column represents the metabolites name.

“Freq” column represents the frequency of the metabolites been significance of the 1,000 times random forest run.

“P” column represents the most significant adjusted p-value of the metabolites from random forest run.

“OR_95 CI” column represents the odds ratio and 95% confidence interval of the correspond p value.

“Tissue” column represents tissue source of the metabolite.

In plasma samples, metabolites such as palmitic acid, phosphatidylethanolamine (PE) (16:1 (11Z)/15:0), ceramide-NS d42:1-sn2, sphingosine-1-phosphate (S1P), and galactose were chosen for model development, as they appeared more than 500 times in random forest models (Table 3). Among these, palmitic acid emerged as the most significant predictor, exhibiting the highest odds ratio (p < 0.003, OR = 5,514.868). The AUC values for the training and test sets were 0.901 and 0.897, respectively, with an overall predictive power of 0.901 (Figure 5C).

Furthermore, an external validation set comprising 75 additional samples was utilized to assess the effectiveness of the predictive model for plasma samples. The validation results showed an AUC value of 0.933 (Figure 5D). The ROC curves for each biomarker were shown in Supplementary Figures 3–5.

### 3.7 Mendelian Randomization verification

A total of ten unique metabolites were included in the MR analyses to assess their potential causal effects on AST and ALT levels (Supplementary Table 1). Among them, 3-hydroxybutyrate and N6-methyllysine showed the most prominent associations. For AST, 3-HB exhibited a significant positive association (Beta = 0.33, 95% CI: 0.11 to 0.56, p = 0.003), suggesting that genetically elevated 3-HB levels may correlate with higher AST. In contrast, for ALT, 3-HB demonstrated a negative estimate (Beta = −0.28, 95% CI: −0.46 to −0.10, p = 2.81e–03), indicating a potentially protective effect. N6-methyllysine was notably associated with ALT, showing a negative relationship (Beta = −0.10, 95% CI: −0.15 to −0.05, p = 6.66e–05), which implies that higher genetically predicted N6-methyllysine may help reduce ALT levels. Other compounds, such as 4-acetamidobutanoate, citrulline, and pyridoxal, displayed nominal or weaker signals, though none reached the same level of significance across both enzymes. Notably, the direction of effects differed for certain metabolites when comparing AST and ALT, suggesting these liver enzymes may be influenced by partially distinct metabolic pathways.

## 4 Discussion

### 4.1 Inter-individual variations are determinants of susceptibility to CIH

Our previous studies demonstrated the significance of individual variations in determining different types of CRAEs (Li et al., 2021a; 2021c; Yao et al., 2022b). These individual differences extend beyond the liver, where CIH develops, and can be influenced by both intrinsic and external factors, such as genetic predispositions, dietary habits, and environmental exposures. Although identifying specific causes is complex, such factors leave distinct imprints on the metabolome. Thus, metabolomic profiling is essential for understanding CIH pathology and developing personalized therapeutic strategies.

In the context of CIH, the contribution weights of collected samples, ranked from lowest to highest, are plasma, CRT, and urine. Interestingly, plasma, despite its direct interaction with liver cells, does not exhibit the highest weight in CIH analysis. This is likely because the blood metabolome plays a central role in maintaining numerous physiological processes, resulting in a narrower range of variation compared to other tissues. For example, the endogenous metabolite hydroxyisopatchoulenone, detected in both plasma and urine (or CRT), displays greater individual variability in urine (coefficient of variation [CV] = 3.93) and CRT (CV = 0.18) compared to plasma (CV = 0.09).

CRT and liver tissues share a common origin from the endodermal germ layer, suggesting a higher degree of similarity in their metabolomic profiles. This makes CRT a promising tissue for identifying potential CIH-associated metabolic traits. Environmental hazards may contribute to CIH and leave detectable metabolic signatures in both CRT and liver tissues. For instance, we observed elevated levels of Cotinine N-oxide in CRT (mean = 1.35, CV = 0.12) compared to plasma (mean = 0.27, CV = 0.04). Cotinine N-oxide is a known cytotoxic compound, underscoring its potential role as a contributing factor to CIH (Nakajima et al., 1998).

Urine, primarily a waste product formed by the kidneys to eliminate excess substances and metabolic byproducts, differs from CRT and plasma in that it does not actively regulate homeostasis or support physiological functions. This characteristic may explain why urine exhibited the highest contribution weight in the CIH analysis. Consistent with previous studies identifying urine-based metabolomic markers for various types of liver injury in humans and animal models, our study is the first to identify urine-specific markers for CIH. This finding highlights the potential of urine as a valuable sample for CIH biomarker discovery.

### 4.2 Potential pathology of CIH

**FIGURE 6:**
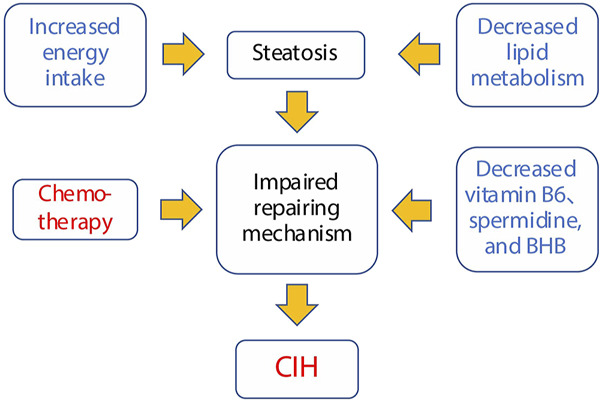
Potential susceptibility factors for chemotherapy-induced hepatotoxicity.

In this subsection, we delve into the comprehensive metabolomic profile associated with CIH. This discussion explores the potential interrelationships among various metabolites and their connection to CIH. Additionally, a summarization of these findings is concisely presented in Figure 6 for a holistic understanding.

#### 4.2.1 Multi-tissue metabolomic profiling indicates steatosis in CIH

In a recent review, four primary chemotherapy-related pathologies associated with liver injury were identified: steatosis, steatohepatitis, sinusoidal obstruction syndrome, and noncirrhotic portal hypertension (Gangi and Lu, 2020b). Our study supports steatosis as the predominant mechanism underlying CIH. Steatosis involves the accumulation of lipids within hepatocytes, which can eventually progress to fibrosis. This condition is commonly observed with chemotherapeutic agents such as capecitabine and oxaliplatin (Gangi and Lu, 2020b).

Lipid accumulation in the liver can be caused by various factors, including increased intake of free fatty acids (FFAs), hindered FFA export from the liver, and decreased FFA oxidation. Consistent with these mechanisms, our analyses reveal a high abundance of lipid-related metabolites in CIH. Specifically, CRT samples indicate a downregulation of lipid metabolism pathways, reflecting suppressed lipid metabolism within the liver. In contrast, plasma samples demonstrate upregulated lipid metabolism pathways, suggesting increased lipid accumulation.

Specifically, plasma exhibited an increased level of galactose, a potential precursor for all lipid molecules through glycolysis (Liu et al., 2000). Galactose is a recognized contributor to liver steatosis, initiating cascades of liver injury (Guo et al., 2018; Sha et al., 2021). As an alternative to glycolysis, galactose can also be converted to galactitol. Its downregulation in CIH provides indirect evidence for upregulated glycolysis and, presumably, lipid accumulation (Tappy, 2021).

Further evidence for steatosis was the significant upregulation of palmitic acid in plasma. As one of the most abundant lipids in the body, palmitic acid serves as a precursor for numerous lipid metabolites critical to cellular functions, including energy storage and intracellular signaling (Han, 2016). In addition, most downstream products of palmitic acid, such as ceramides, phosphatidylethanolamine, and prostaglandin, were downregulated in CIH, further indicating dysregulated lipid metabolism associated with steatosis.

#### 4.2.2 Impaired tissue repair mechanism was the leading risk factor of CIH

Steatosis can give rise to various injuries in splanchnic organs, including inflammation, oxidative stress, and cellular damage. To countermeasure these injuries, an effective tissue repair mechanism is essential. Notably, the abundance and higher MOFA weight of metabolites associated with these injuries suggest impaired tissue repair processes may significantly influence CIH susceptibility.

In the liver, as the body’s primary detoxification organ, it is constantly balancing damage and repair processes. The liver plays a crucial role in metabolizing toxins, drugs, and other harmful substances, making it highly susceptible to injury, especially during chemotherapy. This constant exposure to potential damage requires an efficient repair mechanism to maintain liver function. Vitamin B6, through its involvement in numerous enzymatic reactions essential for amino acid and lipid metabolism, is vital for this repair process. As previously mentioned, insufficient vitamin B6 levels, indicated by lower preoperative levels of 4-pyridoxic acid (Anand, 2005), can impair tissue repair and decrease the liver’s antioxidant capacity, making it more vulnerable to sustained damage (Mitchell et al., 1976). Furthermore, the reduction in M2 macrophage activation (Cabrini et al., 1998; Anand, 2005)., due to vitamin B6 deficiency (Ehmedah et al., 2019), weakens the liver’s ability to repair itself after injury. These combined effects may disrupt the liver’s delicate balance between detoxification, damage, and repair, ultimately contributing to the development of chemotherapy-induced hepatotoxicity (CIH). Therefore, 4-pyridoxic acid and vitamin B6 may serve as critical indicators of liver function and potential risk factors for CIH.

Additionally, elevated levels of the amino acid N6-Methyl-lysine and upregulated amino acid metabolic pathways in CIH patients indicate cytotoxic injury. Previous studies have shown that significant increases in serum and urinary amino acid levels are commonly associated with liver injury (An et al., 2020). Notably, tryptophan, a recognized toxin, was elevated in urine samples and positioned as a central hub in the correlation analysis. Microbial catabolites of tryptophan can bind to the aryl hydrocarbon receptor, activating the immune system and inducing systemic toxic effects (Roager and Licht, 2018). This suggests a potential link between tryptophan dysregulation and steatosis, further contributing to the pathogenesis of CIH.

In CRT samples, patients who developed CIH exhibited lower preoperative levels of BHB, spermidine, adenine, and adenosine. BHB, a ketone body, is known for its significant clinical benefits, particularly in targeting specific organs (Møller, 2020). A ketogenic diet has been shown to prevent hepatic steatosis (Chen Y. et al., 2018), and 3-Hydroxybutyrate exhibits anti-inflammatory and hepatoprotective effects (Choi et al., 2018). Spermidine, a naturally occurring polyamine, has pleiotropic effects, including anti-inflammatory properties, antioxidant functions, and enhancement of mitochondrial metabolic function and respiration (Madeo et al., 2018). Specifically, spermidine can reduce the severity of liver fibrosis and the incidence of hepatocellular carcinoma induced by chemical injury (Yue et al., 2017). Adenine and Adenosine participate in the metabolic pathway of purines, which provide essential components for DNA and RNA. Moreover, purines serve as a source of energy and cofactors necessary for cellular survival and proliferation (Balasubramaniam et al., 2019).

In plasma, patients who developed CIH showed lower S1P levels. S1P, on the other hand, is a phosphosphingolipid shown by Hiroyuki et al. to possess the ability to promote liver cell repair and regeneration, suggesting its potential role in restoring homeostasis following liver injury (Nojima et al., 2016). The study also indicated a reduction in S1P content was associated with liver damage.

Based on the CIH-related metabolic profile and their biological functions, we propose a potential mechanism for CIH (Figure 6). First, impaired tissue repair function is identified as the central susceptibility factor. This dysfunction is linked to several factors, including increased energy intake, which promotes steatosis (fatty liver), and decreased lipid metabolism, further exacerbating lipid accumulation in hepatocytes. Second, reduced levels of critical metabolites such as vitamin B6, spermidine, and BHB impair tissue repair and mitochondrial function, hindering the liver’s ability to regenerate in response to external stimuli. Finally, when chemotherapy is applied, it amplifies this dysfunction, pushing the liver into a state of severe damage, ultimately leading to the development of CIH.

At last, not all detected CIH-related metabolic features exhibited positive validation in the MR analyses, which may be explained by several factors. First, the multifactorial nature of CIH, involving genetic background, lifestyle, and comorbidities, could mean certain metabolites are only relevant in specific patient subgroups. Second, biological complexities, such as context-dependent expression or time-specific fluctuations, may reduce the consistency of detecting these features under the current study design. Lastly, the absence of direct GWAS data for CIH forced us to rely on AST and ALT as proxy indicators of liver injury. Although this interim approach provides initial insights, AST and ALT alone cannot capture the full scope of CIH pathology.

### 4.3 Construction of CIH predictive model

We developed preoperative predictive models for CIH susceptibility using CRT, urine, and plasma metabolic data, all of which demonstrated high predictive performance with AUC values exceeding 0.9. The external validation of the plasma-based model further underscored its clinical utility, achieving an AUC of 0.933. These findings highlight the strong potential of our models in predicting CIH susceptibility prior to surgery. Previous studies primarily focused on plasma samples to evaluate the utility of miRNAs as CIH predictive biomarkers (Hwang et al., 2023; Liu et al., 2023). In contrast, our approach incorporated multi-tissue metabolomic data, enhancing predictive accuracy. Compared to other machine learning or deep learning-based models, such as those in Rao et al. (2023), which employed penalized logistic regression (AUC = 0.88), neural networks (AUC = 0.87), random forests (AUC = 0.85), support vector machines (AUC = 0.85), weighted averages (AUC = 0.88), and ensemble learning (AUC = 0.88), our models demonstrated superior performance, achieving AUC values above 0.9. (Rao et al., 2023). Notably, our models leveraged pre-surgery samples to predict CIH susceptibility, whereas many previous studies utilized features collected post-CIH onset (Guan et al., 2022). Building on our earlier work, such as predictive models for hand-foot syndrome (Li et al., 2021b) and thrombocytopenia (Yao et al., 2022a), we introduced a plasma-targeted metabolites model validated externally, achieving an AUC exceeding 0.93. This addition further strengthens the reliability of our approach. However, the model’s performance is limited by the relatively small dataset used for training and validation. Further studies with larger external test datasets are necessary to confirm the generalizability of the urine and CRT models, ensuring their robustness across diverse patient populations.

## 5 Conclusion

This study employed untargeted metabolomics to identify key compounds associated with CIH across CRT, plasma, and urine samples. The results highlight disturbances in polyamine, lipid, and purine metabolism, along with reduced 4-pyridoxic acid levels, as critical susceptibility factors for CIH. These metabolic disruptions, coupled with diminished cellular repair functions, represent endogenous mechanisms contributing to hepatotoxicity.

There are certain limitations in our study that should be addressed. Firstly, the sample size used in both the discovery and validation phases was relatively small, which may limit the generalizability and robustness of the predictive models. Larger, multi-center cohorts with more diverse patient populations are necessary to validate the results and enhance the reliability of the findings. Secondly, while our study focused on metabolomics, it did not incorporate other omics layers, such as genomics, transcriptomics, or proteomics. Integrating these additional omics data would provide a more comprehensive understanding of the molecular mechanisms underlying chemotherapy-induced hepatotoxicity (CIH) and strengthen the predictive accuracy of our models. Moreover, our analysis was based on plasma, urine, and colorectal tissue samples, and including liver tissue directly would allow for more liver-specific insights. Finally, the study’s cross-sectional design limits our ability to assess the temporal dynamics of metabolic changes in response to chemotherapy. Longitudinal studies combined with multi-omics integration are essential for a deeper understanding of how these metabolic alterations evolve over time and contribute to CIH development.

## Data Availability

The raw data supporting the conclusions of this article will be made available by the authors, without undue reservation.

## References

[B1] An Z. Hu T. Lv Y. Li P. Liu L. (2020). Targeted amino acid and related amines analysis based on iTRAQ®-LC-MS/MS for discovering potential hepatotoxicity biomarkers. J. Pharm. Biomed. Anal. 178, 112812. 10.1016/j.jpba.2019.112812 31639596

[B2] Anand S. S. (2005). Protective effect of vitamin B6 in chromium-induced oxidative stress in liver. J. Appl. Toxicol. 25, 440–443. 10.1002/jat.1077 15986493

[B3] Argelaguet R. Arnol D. Bredikhin D. Deloro Y. Velten B. Marioni J. C. (2020). MOFA+: a statistical framework for comprehensive integration of multi-modal single-cell data. Genome Biol. 21, 111. 10.1186/s13059-020-02015-1 32393329 PMC7212577

[B4] Argelaguet R. Velten B. Arnol D. Dietrich S. Zenz T. Marioni J. C. (2018). Multi‐Omics Factor Analysis—a framework for unsupervised integration of multi‐omics data sets. Mol. Syst. Biol. 14, e8124. 10.15252/msb.20178124 29925568 PMC6010767

[B5] Aubrecht J. Schomaker S. J. Amacher D. E. (2013). Emerging hepatotoxicity biomarkers and their potential to improve understanding and management of drug-induced liver injury. Genome Med. 5, 85–93. 10.1186/gm489 24073687 PMC3979132

[B6] Balasubramaniam S. Christodoulou J. Rahman S. (2019). Disorders of riboflavin metabolism. J. Inherit. Metab. Dis. 42, 608–619. 10.1002/jimd.12058 30680745

[B7] Bijlsma S. Bobeldijk I. Verheij E. R. Ramaker R. Kochhar S. Macdonald I. A. (2006). Large-scale human metabolomics studies: a strategy for data (pre-) processing and validation. Anal. Chem. 78, 567–574. 10.1021/ac051495j 16408941

[B8] Bray F. Ferlay J. Soerjomataram I. Siegel R. L. Torre L. A. Jemal A. (2018). Global cancer statistics 2018: GLOBOCAN estimates of incidence and mortality worldwide for 36 cancers in 185 countries. CA Cancer J. Clin. 68, 394–424. 10.3322/caac.21492 30207593

[B9] Bujak R. Struck-Lewicka W. Markuszewski M. J. Kaliszan R. (2015). Metabolomics for laboratory diagnostics. J. Pharm. Biomed. Anal. 113, 108–120. 10.1016/j.jpba.2014.12.017 25577715

[B10] Cabrini L. Bergami R. Fiorentini D. Marchetti M. Landi L. Tolomelli B. (1998). Vitamin B6 deficiency affects antioxidant defences in rat liver and heart. Biochem. Mol. Biol. Int. 46, 689–697. 10.1080/15216549800204222 9844729

[B11] Chen J. Qi Y. Zhao Y. Kaczorowski D. Couttas T. A. Coleman P. R. (2018a). Deletion of sphingosine kinase 1 inhibits liver tumorigenesis in diethylnitrosamine-treated mice. Oncotarget 9, 15635–15649. 10.18632/oncotarget.24583 29643998 PMC5884653

[B12] Chen Y. Ouyang X. Hoque R. Garcia-Martinez I. Yousaf M. N. Tonack S. (2018b). β-Hydroxybutyrate protects from alcohol-induced liver injury via a Hcar2-cAMP dependent pathway. J. Hepatol. 69, 687–696. 10.1016/j.jhep.2018.04.004 29705237 PMC6098974

[B13] Choi H.-R. Kim J. Lim H. Park Y. K. (2018). Two-week exclusive Supplementation of Modified ketogenic Nutrition Drink Reserves lean body mass and Improves blood lipid profile in obese Adults: a Randomized clinical trial. Nutrients 10, 1895. 10.3390/nu10121895 30513970 PMC6316485

[B14] Ehmedah A. Nedeljkovic P. Dacic S. Repac J. Draskovic Pavlovic B. Vucevic D. (2019). Vitamin B complex treatment Attenuates local inflammation after Peripheral Nerve injury. Molecules 24, 4615. 10.3390/molecules24244615 31861069 PMC6943485

[B15] Everett J. R. Holmes E. Veselkov K. A. Lindon J. C. Nicholson J. K. (2019). A Unified Conceptual framework for metabolic phenotyping in diagnosis and prognosis. Trends Pharmacol. Sci. 40, 763–773. 10.1016/j.tips.2019.08.004 31511194

[B16] Gangi A. Lu S. C. (2020a). Chemotherapy-associated liver injury in colorectal cancer. Ther. Adv. Gastroenterol. 13, 1756284820924194. 10.1177/1756284820924194 PMC7249601 32547639

[B17] Gangi A. Lu S. C. (2020b). Chemotherapy-associated liver injury in colorectal cancer. Ther. Adv. Gastroenterol. 13, 1756284820924194. 10.1177/1756284820924194 PMC7249601 32547639

[B18] Gelibter A. J. Caponnetto S. Urbano F. Emiliani A. Scagnoli S. Sirgiovanni G. (2019). Adjuvant chemotherapy in resected colon cancer: when, how and how long? Surg. Oncol. 30, 100–107. 10.1016/j.suronc.2019.06.003 31500770

[B19] Gillespie M. Jassal B. Stephan R. Milacic M. Rothfels K. Senff-Ribeiro A. (2022). The reactome pathway knowledgebase 2022. Nucleic Acids Res. 50, D687–D692. 10.1093/nar/gkab1028 34788843 PMC8689983

[B20] Guan S. Chen X. Chen Y. Wan G. Su Q. Liang H. (2022). FOXO3 mutation predicting gefitinib-induced hepatotoxicity in NSCLC patients through regulation of autophagy. Acta Pharm. Sin. B 12, 3639–3649. 10.1016/j.apsb.2022.02.006 36176901 PMC9513443

[B21] Guo C. Chen L. Huang J. Wang Y. Shi C. Gao J. (2018). Aldose reductase inhibitor protects mice from alcoholic steatosis by repressing saturated fatty acid biosynthesis. Chem. Biol. Interact. 287, 41–48. 10.1016/j.cbi.2018.04.002 29630881

[B22] Han X. (2016). Lipidomics for studying metabolism. Nat. Rev. Endocrinol. 12, 668–679. 10.1038/nrendo.2016.98 27469345

[B23] Hwang D.-B. Seo Y. Lee E. Won D.-H. Kim C. Kang M. (2023). Diagnostic potential of serum miR-532-3p as a circulating biomarker for experimental intrinsic drug-induced liver injury by acetaminophen and cisplatin in rats. Food Chem. Toxicol. 178, 113890. 10.1016/j.fct.2023.113890 37308052

[B24] Ji Y. Chen H. Gow W. Ma L. Jin Y. Hui B. (2020). Potential biomarkers Ang II/AT1R and S1P/S1PR1 predict the prognosis of hepatocellular carcinoma. Oncol. Lett. 20 (5), 208–211. 10.3892/ol.2020.12071 32963614 PMC7491028

[B25] Li M. Chen J. Deng Y. Yan T. Gu H. Zhou Y. (2021a). Risk prediction models based on hematological/body parameters for chemotherapy-induced adverse effects in Chinese colorectal cancer patients. Support Care Cancer 29, 7931–7947. 10.1007/s00520-021-06337-z 34213641

[B26] Li M. Chen J. Liu S. Sun X. Xu H. Gao Q. (2021b). Spermine-related DNA Hypermethylation and elevated expression of Genes for Collagen formation are susceptible factors for chemotherapy-induced hand-foot syndrome in Chinese colorectal cancer patients. Front. Pharmacol. 12, 746910. 10.3389/fphar.2021.746910 34539419 PMC8440935

[B27] Li M. Chen J. Liu S. Sun X. Xu H. Gao Q. (2021c). Spermine-related DNA Hypermethylation and elevated expression of Genes for Collagen formation are susceptible factors for chemotherapy-induced hand-foot syndrome in Chinese colorectal cancer patients. Front. Pharmacol. 12, 746910. 10.3389/fphar.2021.746910 34539419 PMC8440935

[B28] Liu G. Hale G. E. Hughes C. L. (2000). Galactose metabolism and ovarian toxicity. Reprod. Toxicol. 14, 377–384. 10.1016/s0890-6238(00)00096-4 11020650

[B29] Liu Y. Guan H. Feng M. Du C. Zhang Q. Shou Y. (2023). MiR-766-3p and miR-671-5p attenuate aristolochic acid-induced hepatotoxicity by directly targeting the key bioactivating enzyme NQO1. Ecotoxicol. Environ. Saf. 261, 115103. 10.1016/j.ecoenv.2023.115103 37285672

[B30] Madeo F. Eisenberg T. Pietrocola F. Kroemer G. (2018). Spermidine in health and disease. Science 359, eaan2788. 10.1126/science.aan2788 29371440

[B31] McDonald G. B. Tirumali N. (1984). Intestinal and liver toxicity of antineoplastic drugs. West J. Med. 140, 250–259. 6375139 PMC1021607

[B32] McGill M. R. Jaeschke H. (2019). Animal models of drug-induced liver injury. Biochim. Biophys. Acta Mol. Basis Dis. 1865, 1031–1039. 10.1016/j.bbadis.2018.08.037 31007174 PMC6478394

[B33] McWhirter D. Kitteringham N. Jones R. P. Malik H. Park K. Palmer D. (2013). Chemotherapy induced hepatotoxicity in metastatic colorectal cancer: a review of mechanisms and outcomes. Crit. Rev. Oncol. Hematol. 88, 404–415. 10.1016/j.critrevonc.2013.05.011 23786843

[B34] Meunier L. Larrey D. (2019). Drug-induced liver injury: biomarkers, Requirements, candidates, and validation. Front. Pharmacol. 10, 1482. 10.3389/fphar.2019.01482 31920666 PMC6917655

[B35] Mitchell D. Wagner C. Stone W. J. Wilkinson G. R. Schenker S. (1976). Abnormal regulation of plasma pyridoxal 5’-phosphate in patients with liver disease. Gastroenterology 71, 1043–1049. 10.1016/s0016-5085(76)80056-x 992265

[B36] Møller N. (2020). Ketone body, 3-hydroxybutyrate: Minor metabolite - Major Medical manifestations. J. Clin. Endocrinol. Metab. 105, dgaa370. 10.1210/clinem/dgaa370 32525972

[B37] Nakajima M. Iwata K. Yamamoto T. Funae Y. Yoshida T. Kuroiwa Y. (1998). Nicotine metabolism in liver microsomes from rats with acute hepatitis or cirrhosis. Drug Metab. Dispos. 26, 36–41. 9443850

[B38] Nojima H. Freeman C. M. Schuster R. M. Japtok L. Kleuser B. Edwards M. J. (2016). Hepatocyte exosomes mediate liver repair and regeneration via sphingosine-1-phosphate. J. Hepatol. 64, 60–68. 10.1016/j.jhep.2015.07.030 26254847 PMC4843792

[B39] Rao M. Nassiri V. Alhambra C. Snoeys J. Van Goethem F. Irrechukwu O. (2023). AI/ML models to predict the severity of drug-induced liver injury for small molecules. Chem. Res. Toxicol. 36, 1129–1139. 10.1021/acs.chemrestox.3c00098 37294641

[B40] Rinschen M. M. Ivanisevic J. Giera M. Siuzdak G. (2019). Identification of bioactive metabolites using activity metabolomics. Nat. Rev. Mol. Cell Biol. 20, 353–367. 10.1038/s41580-019-0108-4 30814649 PMC6613555

[B41] Ritchie M. E. Phipson B. Wu D. Hu Y. Law C. W. Shi W. (2015). Limma powers differential expression analyses for RNA-sequencing and microarray studies. Nucleic Acids Res. 43, e47. 10.1093/nar/gkv007 25605792 PMC4402510

[B42] Roager H. M. Licht T. R. (2018). Microbial tryptophan catabolites in health and disease. Nat. Commun. 9, 3294. 10.1038/s41467-018-05470-4 30120222 PMC6098093

[B43] Sha J.-Y. Li J.-H. Zhou Y.-D. Yang J.-Y. Liu W. Jiang S. (2021). The p53/p21/p16 and PI3K/Akt signaling pathways are involved in the ameliorative effects of maltol on D-galactose-induced liver and kidney aging and injury. Phytother. Res. 35, 4411–4424. 10.1002/ptr.7142 34028092

[B44] Shen R. Mo Q. Schultz N. Seshan V. E. Olshen A. B. Huse J. (2012). Integrative subtype discovery in glioblastoma using iCluster. PLoS One 7, e35236. 10.1371/journal.pone.0035236 22539962 PMC3335101

[B45] Shen R. Olshen A. B. Ladanyi M. (2009). Integrative clustering of multiple genomic data types using a joint latent variable model with application to breast and lung cancer subtype analysis. Bioinformatics 25, 2906–2912. 10.1093/bioinformatics/btp543 19759197 PMC2800366

[B46] Tappy L. (2021). Metabolism of sugars: a window to the regulation of glucose and lipid homeostasis by splanchnic organs. Clin. Nutr. 40, 1691–1698. 10.1016/j.clnu.2020.12.022 33413911

[B47] Weiss R. H. Kim K. (2011). Metabolomics in the study of kidney diseases. Nat. Rev. Nephrol. 8, 22–33. 10.1038/nrneph.2011.152 22025087

[B48] Yang K. Woodhead J. L. Watkins P. B. Howell B. A. Brouwer K. L. R. (2014). Systems Pharmacology modeling predicts Delayed Presentation and Species differences in bile acid–Mediated Troglitazone hepatotoxicity. Clin. Pharmacol. & Ther. 96, 589–598. 10.1038/clpt.2014.158 25068506 PMC4480860

[B49] Yao H. Xu H. Qiu S. Chen J. Lin Z. Zhu J. (2022a). Choline deficiency-related multi-omics characteristics are susceptible factors for chemotherapy-induced thrombocytopenia. Pharmacol. Res. 178, 106155. 10.1016/j.phrs.2022.106155 35248699

[B50] Yao H. Xu H. Qiu S. Chen J. Lin Z. Zhu J. (2022b). Choline deficiency-related multi-omics characteristics are susceptible factors for chemotherapy-induced thrombocytopenia. Pharmacol. Res. 178, 106155. 10.1016/j.phrs.2022.106155 35248699

[B51] Yue F. Li W. Zou J. Jiang X. Xu G. Huang H. (2017). Spermidine Prolongs Lifespan and prevents liver fibrosis and hepatocellular carcinoma by activating MAP1S-Mediated autophagy. Cancer Res. 77, 2938–2951. 10.1158/0008-5472.CAN-16-3462 28386016 PMC5489339

[B52] Zhang S. Li Q. Liu J. Zhou X. J. (2011). A novel computational framework for simultaneous integration of multiple types of genomic data to identify microRNA-gene regulatory modules. Bioinformatics 27, i401–i409. 10.1093/bioinformatics/btr206 21685098 PMC3117336

[B53] Zhang S. Liu C.-C. Li W. Shen H. Laird P. W. Zhou X. J. (2012). Discovery of multi-dimensional modules by integrative analysis of cancer genomic data. Nucleic Acids Res. 40, 9379–9391. 10.1093/nar/gks725 22879375 PMC3479191

[B54] Zheng Y. Liu Y. Yang J. Dong L. Zhang R. Tian S. (2023). Multi-omics data integration using ratio-based quantitative profiling with Quartet reference materials. Nat. Biotechnol. 42, 1133–1149. 10.1038/s41587-023-01934-1 37679543 PMC11252085

