# Supplementary material for: Multi-tissue metabolomics analysis reveals susceptible factors for chemotherapy-induced hepatotoxicity in colorectal cancer patients
Source: Front Pharmacol. 2025 Apr 4;16:1517446. doi: 10.3389/fphar.2025.1517446 (PMC12006014; doi:10.3389/fphar.2025.1517446)
Supplement: Supplementary file 2 [file Supplementaryfile1.docx]

**Supplementary Figures**

**Supplementary Figure 1. Mendelian Randomization Analysis of Metabolite Levels and Alanine Aminotransferase (ALT) Levels**

**The results of Mendelian Randomization (MR) analysis investigating the causal relationships between various metabolite levels (Exposure) and Aspartate Aminotransferase (ALT) levels.**

nSNP: Number of SNPs used in the MR analysis.

Beta (95% CI): Effect size estimate and its 95% confidence interval, indicating the predicted influence of metabolite levels on AST levels.

P-value: Statistical significance of the association. Red values highlight significant associations (P < 0.05).

**Supplementary Figure 2. Mendelian Randomization Analysis of Metabolite Levels and Aspartate Aminotransferase (AST) Levels**

**The results of Mendelian Randomization (MR) analysis investigating the causal relationships between various metabolite levels (Exposure) and Aspartate Aminotransferase (AST) levels.**

nSNP: Number of SNPs used in the MR analysis.

Beta (95% CI): Effect size estimate and its 95% confidence interval, indicating the predicted influence of metabolite levels on AST levels.

P-value: Statistical significance of the association. Red values highlight significant associations (P < 0.05).

**Supplementary Figure 3. ROC Curve for each biomarker of the Prediction Model for CRT.**

**
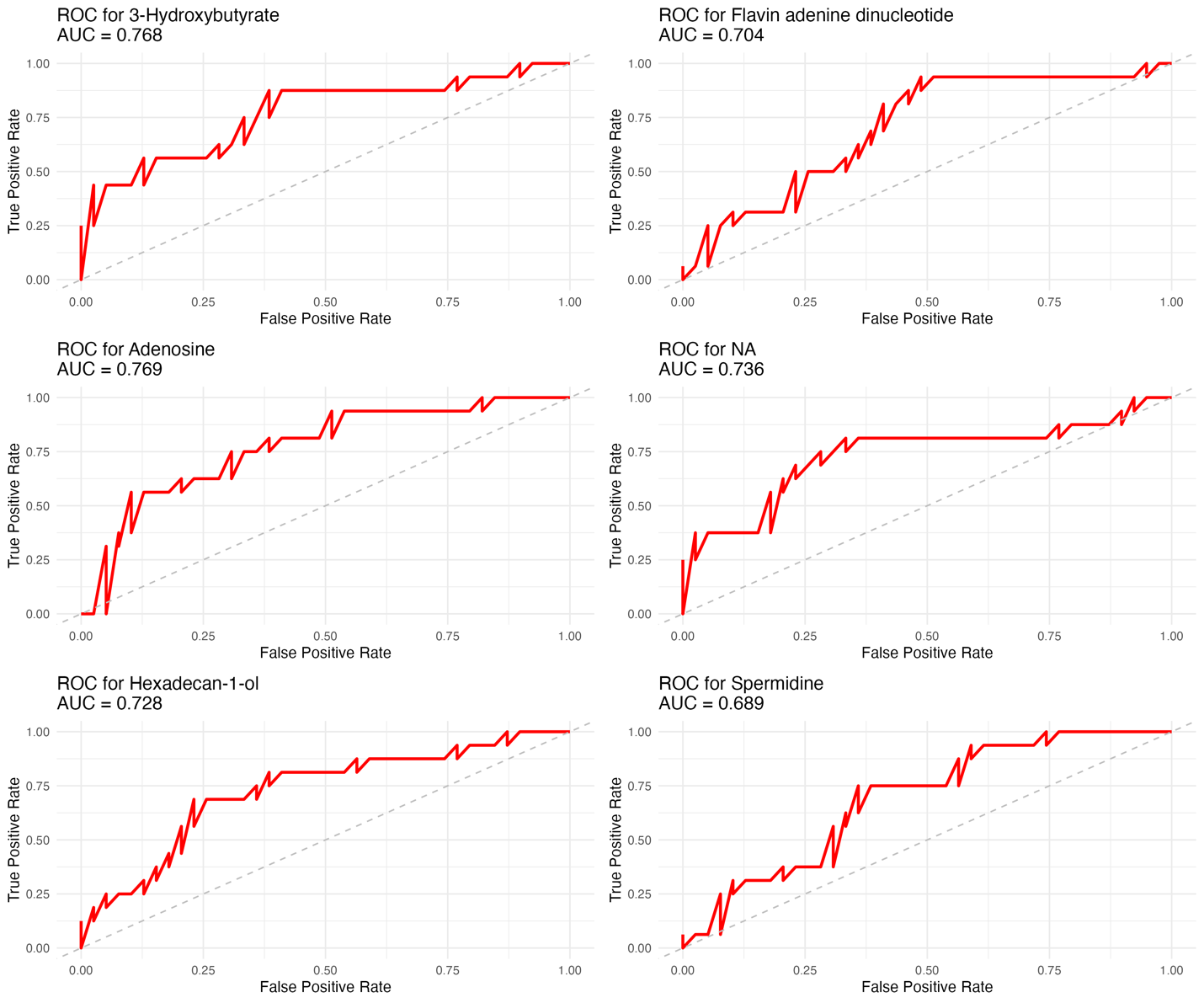
**

**Supplementary Figure 4. ROC Curve for each biomarker of the Prediction Model for Plasma.**

**
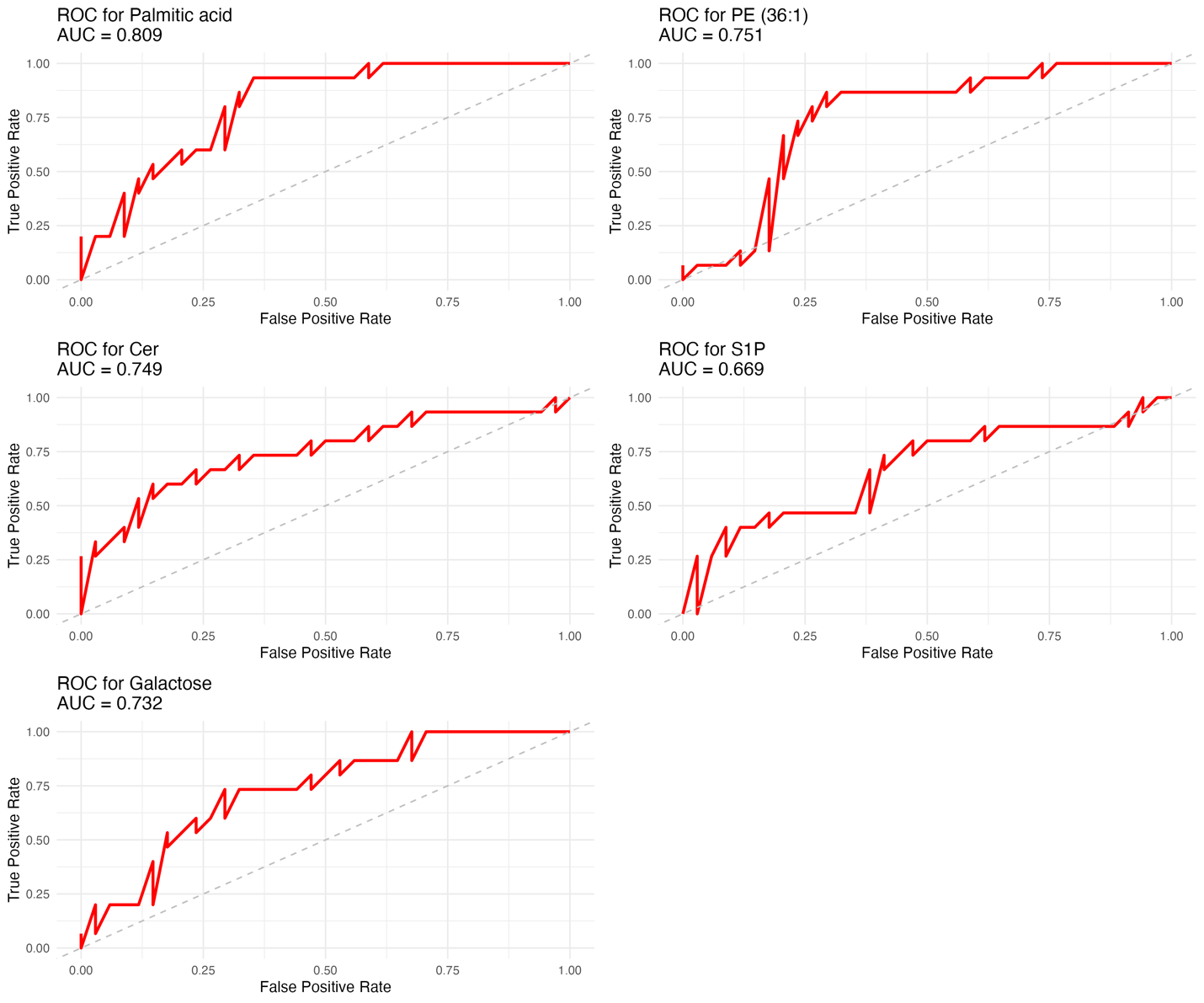
**

**Supplementary Figure 5. ROC Curve for each biomarker of the Prediction Model for Urine.**

**
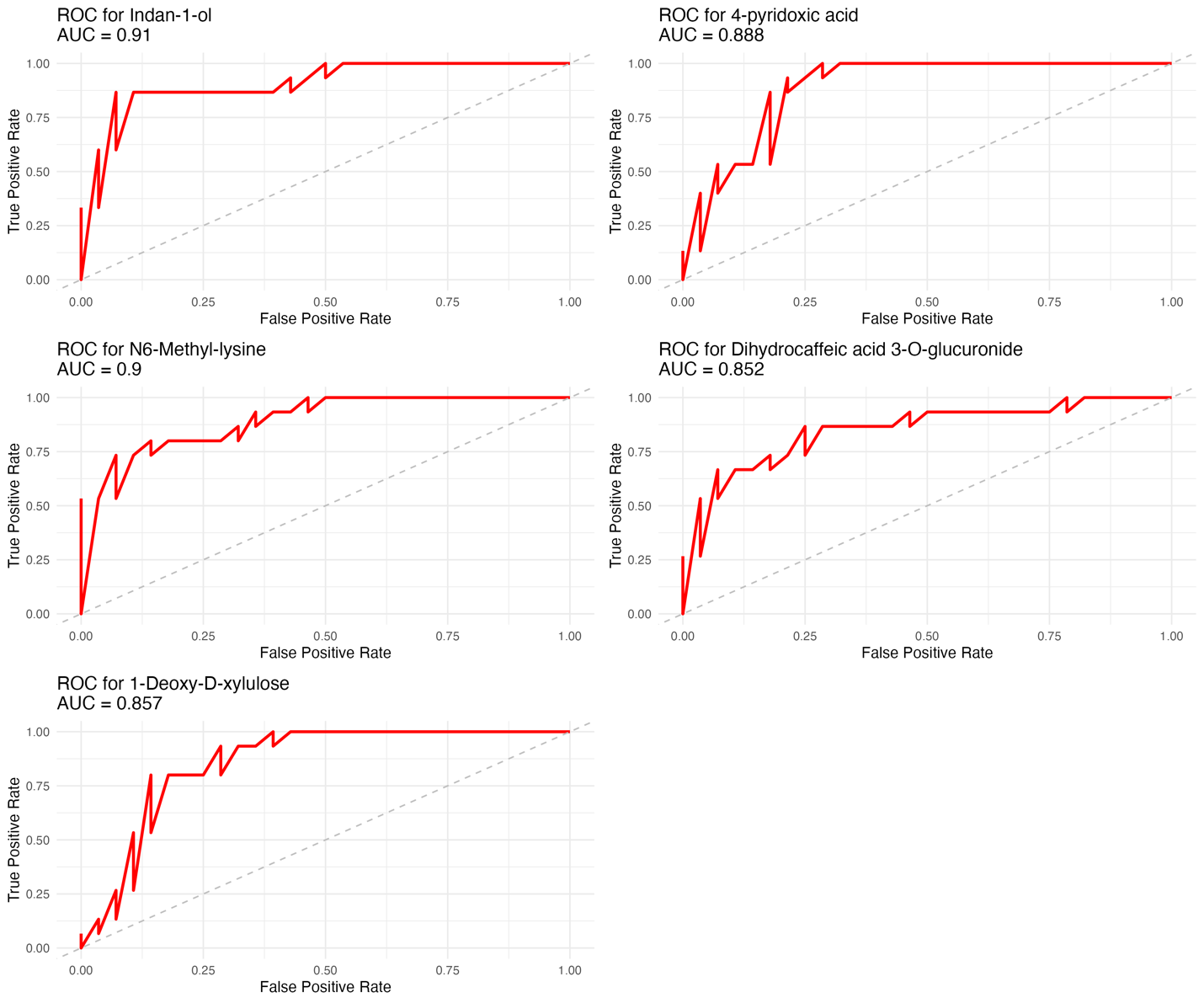
**

**Supplementary Table 3. Updated mass spectrums of identified metabolites at first revision.** For each metabolite identified by standard, the upper figure in each panel shows the spectrum from urine and lower panel shows the spectrum from commercial standards. Compound spectra 1, 2, 10, 19, 30 are obtained by comparison of our experiments with standards.

| **Name** | **HMDB** | **Tissue** | **Electrospray Ionization Mode** | **Mass spectrum of identified metabolites** |
| --- | --- | --- | --- | --- |
| 3-Hydroxybutyric acid | HMDB0000011 | CRT | negative | 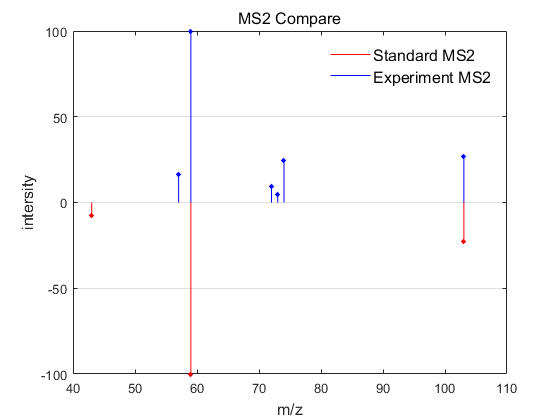 |
| Flavin mononucleotide | HMDB0001520 | CRT | positive |  |
| Hexadecan-1-ol | HMDB0003424 | CRT | positive |  |
| Adenine | HMDB0000034 | CRT | negative | 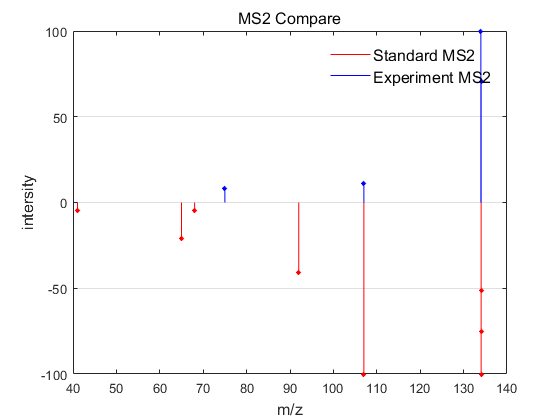 |
| Spermidine | HMDB0001257 | CRT | positive |  |
| Adenosine | HMDB0000050 | CRT | positive | 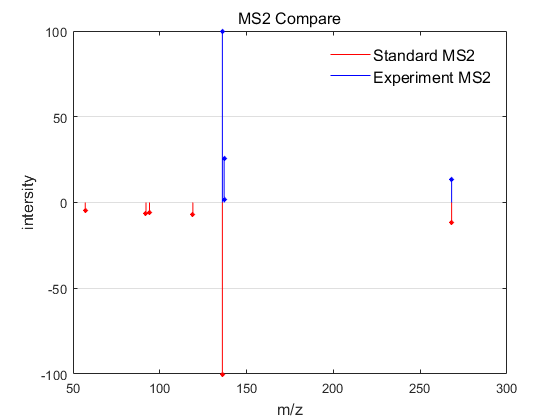 |
| Prostaglandin F2α | HMDB0001139 | CRT | negative |  |
| PE (36:1) | HMDB0008992 | Plasma | positive |  |
| PE (36:1) | HMDB0008992 | Plasma | positive | 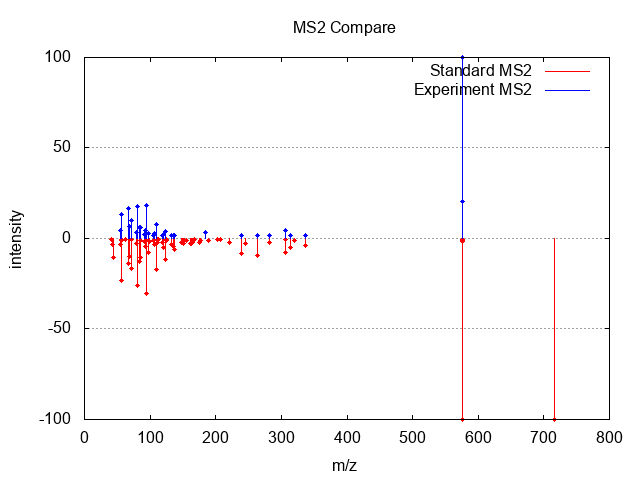 |
| LXA4 (Lipoxin A4) | HMDB0004385 | Plasma | positive |  |
| LXA4 (Lipoxin A4) | HMDB0004385 | Plasma | negative |  |
| Palmitic acid | HMDB0000220 | Plasma | negative | 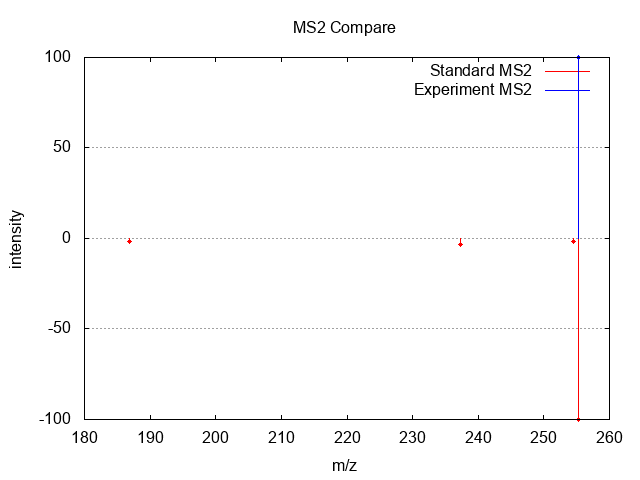 |
| Palmitic acid | HMDB0000220 | Plasma | positive | 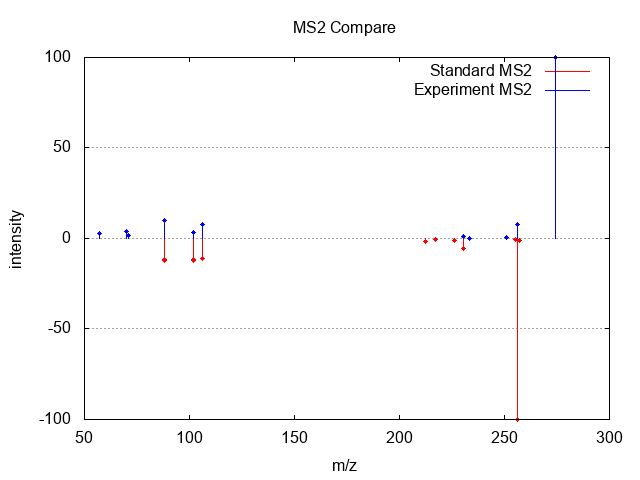 |
| S1P | HMDB0000277 | Plasma | negative | 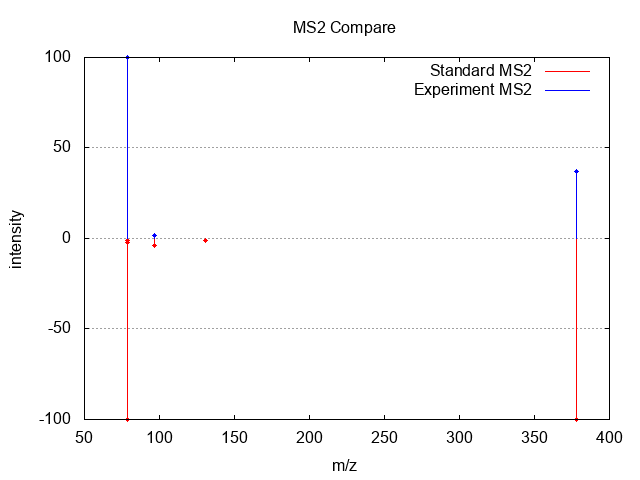 |
| Tyrosine | HMDB0000158 | Plasma | negative |  |
| Tyrosine | HMDB0000158 | Plasma | positive | 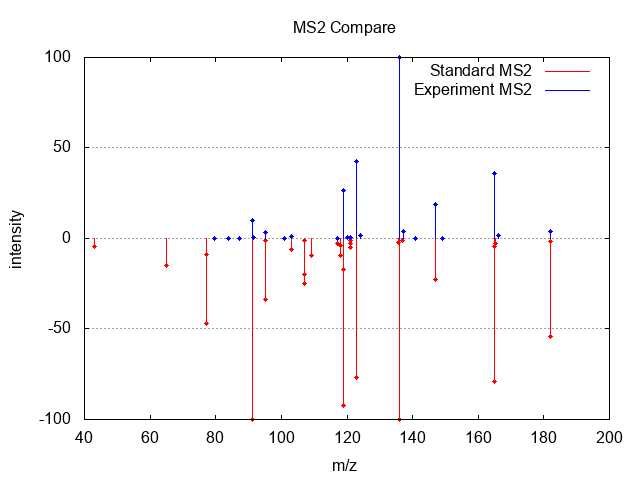 |
| Cer (Cer-NS d34:1) | HMDB0004949 | Plasma | negative | 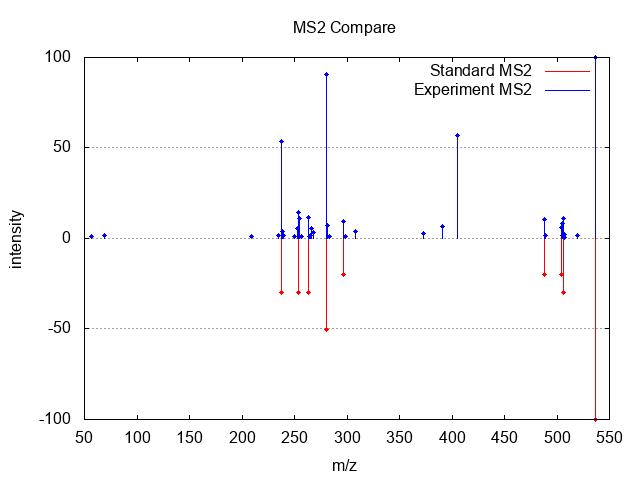 |
| Galactose | HMDB0000143 | Plasma | negative | 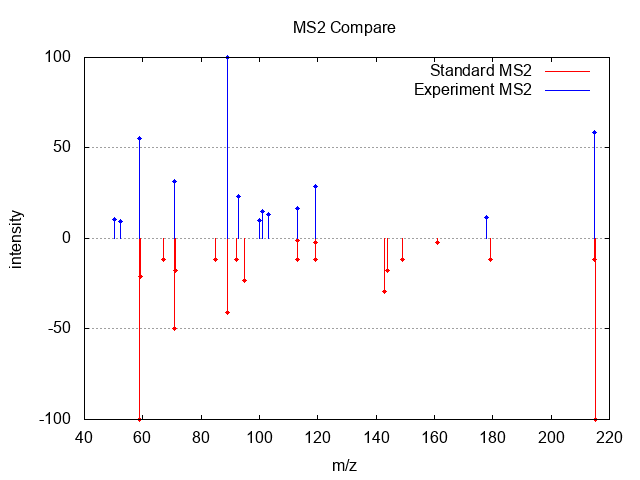 |
| Tryptophan | HMDB0000929 | Urine | positive |  |
| 1-Deoxy-D-xylulose | HMDB0001292 | Urine | negative |  |
| 4-Acetamidobutanoate | HMDB0003681 | Urine | negative | 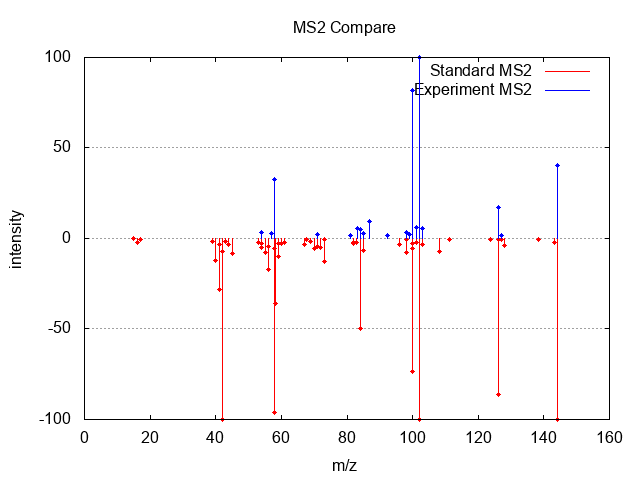 |
| 4-Pyridoxic acid | HMDB0000017 | Urine | negative |  |
| 4-Pyridoxic acid | HMDB0000017 | Urine | positive |  |
| Hypoxanthine | HMDB0000157 | Urine | positive |  |
| 2-Hydroxy-2-methylbutanenitrile | HMDB0060309 | Urine | positive |  |
| N6-Methyl-lysine | HMDB0002038 | Urine | positive |  |
| Tyrosine | HMDB0000158 | Urine | positive | 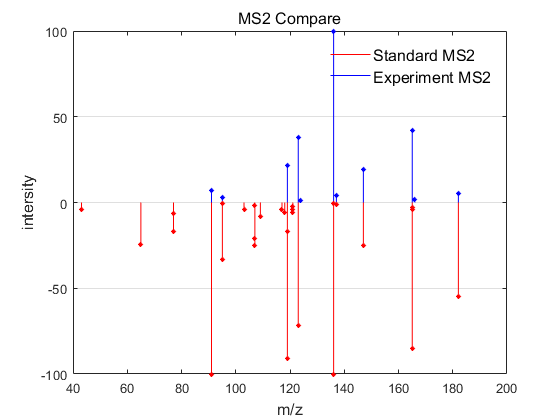 |
| Indan-1-ol | HMDB0059601 | Urine | positive |  |
| Leucine | HMDB0000687 | Urine | positive |  |
| Glutamine | HMDB0003423 | Urine | positive | 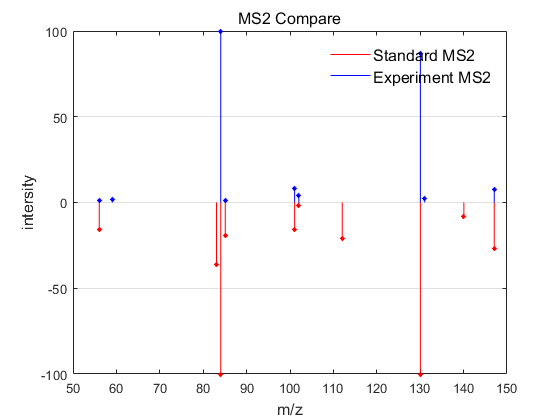 |
| 2,3-Methylene suberic acid | HMDB0059779 | Urine | negative | 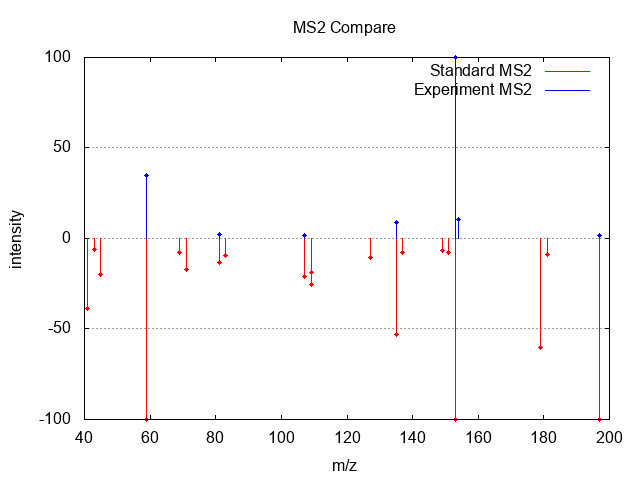 |
| Citrulline | HMDB0000904 | Urine | negative |  |
| Dihydrocaffeic acid 3-O-glucuronide | HMDB0041720 | Urine | negative |  |
| Galactitol | HMDB0000107 | Urine | negative |  |
| 3-Oxopropanoate | HMDB0011111 | Urine | negative |  |
